# Evaluation of phytotoxic potential in Asteraceae plant extracts for biological control of *Echinochloa crus‐galli* and *Echinochloa colona*

**DOI:** 10.1002/pei3.70009

**Published:** 2024-09-09

**Authors:** Nguyen Thi Thuy Trang, Nguyen The Cuong, Le Van Vang, Ho Le Thi

**Affiliations:** ^1^ Plant Protection Faculty College of Agriculture, Can Tho University Can Tho Vietnam; ^2^ Cuu Long Delta Rice Research Institute Can Tho Vietnam

**Keywords:** allelopathy, Asteraceae, *Echinochloa colona* L*.*, *Echinochloa crus‐galli* L., HPLC

## Abstract

This study evaluates the phytotoxic potential of methanolic extracts from six Asteraceae species: *Wedelia chinensis*, *Helianthus annuus*, *Cosmos bipinnatus*, *Tagetes erecta*, *Tithonia diversifolia*, *and Zinnia elegans*. The extracts were tested at concentrations of 0.03, 0.1, 0.3, and 1.0 g/mL to assess their inhibitory effects on the radicle and hypocotyl lengths of *Echinochloa crus‐galli* and *Echinochloa colona*. The two most potent species, *C. bipinnatus* and *T. diversifolia*, were further evaluated using extracts from their roots, stems, and leaves. Among these, *C. bipinnatus* leaf extracts showed the most significant phytotoxicity and were tested at 20, 40, and 60 days of plant age. At 0.03 g/mL, *C. bipinnatus* extract inhibited the hypocotyl and radicle lengths of *E. colona* by 23.01% and 56.45%, and *E. crus‐galli* by 8.5% and 36.35%, respectively. At 1.0 g/mL, the extract inhibited the hypocotyl lengths of *E. colona* and *E. crus‐galli* by 97.54% and 88.15%, and the radicle lengths by 93.52% and 99.99%, respectively. The 60‐day‐old *C. bipinnatus* leaf extract exhibited the highest inhibitory effect, correlating with the identification of key allelochemicals such as cinnamic acid, caffeic acid, coumaric acid, ferulic acid, 2‐4 dimethohydroxy benzoic acid, and salicylic acid. These findings suggest that the 60‐day‐old *C. bipinnatus* leaf extracts have strong potential for use in the biological control of these weed species, offering a promising avenue for the development of natural herbicides.

## Graphical abstract

This study evaluated the phytotoxic potential of methanol extracts from six Asteraceae plants against *Echinochloa crus‐galli* and *Echinochloa colona*. *Cosmos bipinnatus* extract exhibited the most stable inhibition, with significant effects on radicle and hypocotyl lengths of both weeds. The 60‐day‐old *C. bipinnatus* leaf extract, rich in compounds like cinnamic acid and caffeic acid, showed the highest inhibitory effect, suggesting its potential for bioherbicide development against *E. crus‐galli* and *E. colona*.

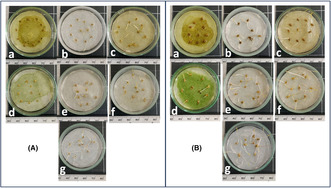

## 1 INTRODUCTION

*Echinochloa crus‐galli* (barnyard grass) and *Echinochloa colona* (jungle rice) are highly invasive weed species that significantly threaten global rice production. Their rapid growth, high seed production, and adaptability to diverse conditions allow them to outcompete rice plants for resources, leading to yield losses of up to 80% (Rao, 2021). The use of synthetic herbicides has caused many negative effects on the environment, including water pollution, soil degradation, and loss of biodiversity. Their widespread herbicide resistance further complicates management efforts (Damalas & Koutroubas, 2023; Heap, 2014). For instance, *E. crus‐galli* in Australia (Gaines et al., 2012) and California (Alarcón‐Reverte et al., 2017) has developed herbicide resistance to glyphosate. Wright et al. (2018) isolated genes that confer resistance to four herbicides: imazamox, fenoxaprop‐P‐ethyl, quinclorac, and propanil. Cyhalofop‐butyl is a herbicide that specifically inhibits *Echinochloa*. Spp. and is also reported to be resistant to *E. colona* in rice fields and under conditions of high CO_2_ temperature (Refatti et al., 2019). In Brazil, *E. crus‐galli* has shown resistance to imidazolinone in 81% of samples and quinclorac in 19% of samples (Matzenbacher et al., 2015). A study by Chen et al. (2016) found that *E. crus‐galli* is resistant to several herbicides, including bispyribac sodium, quinclorac, and metamifop. Integrated weed management (IWM) strategies, combining cultural, mechanical, and biological methods, are critical in addressing these challenges (Bastiaans et al., 2008). Recent studies underscore the urgent need for sustainable weed management alternatives, such as bioherbicides derived from secondary metabolites of plants (Respatie et al., 2019). Secondary metabolites from plants may be able to replace synthetic herbicides because natural products from plants have the potential to inhibit the growth and development of weeds (Musyimi et al., 2012; Respatie et al., 2019). In addition, some studies have found that allelopathic plant extracts can be used as natural herbicides to control weeds (Musa et al., 2017; Narwal, 1999; Thi et al., 2022). Sustainable approaches are essential to mitigate the impact of *Echinochloa* species on rice yields, ensuring agricultural productivity and environmental protection (Rao, 2021).

Asteraceae family plants, also known as Compositae, are one of the largest and most diverse families of flowering plants. Many species of Asteraceae plants have been found to possess allelopathic properties, which can inhibit the growth and development of otcher plant species (Campbell et al., 1982). Recent studies have shown that the family contains allelopathic compounds that can be used as bioherbicides. For instance, *H. annuus*, commonly known as Mexican sunflower, is well documented for its allelopathic effects (Ashrafi et al., 2010; Leather, 1983; Macías et al., 2002). It has also revealed that incorporating sunflower residues into the soil significantly reduced both the total number and biomass of weeds in the wheat field (Alsaadawi et al., 2011). Research by Oke et al. (2011) indicated that aqueous extracts from *T. diversifolia* leaves significantly reduced the growth of seedlings of *Monodora tenuifolia*, *Dialium guineense*, and *Hildegardia barteri*. Nawaz et al. (2013) reported that *Eupatorium odoratum* extract inhibits the growth of *Zea mays* L. and other crops. Similarly, studies by Rawat et al. (2017) demonstrated the allelopathic effects of *Helianthus annuus* on *Amaranthus retroflexus* and *Digitaria sanguinalis*. Moreover, research by Laosinwattana et al. (2018) identified that *Tagetes erecta* contains terpenoids that have strong inhibitory effects on *Echinochloa crus‐galli*. Respatie et al. (2019) found that *Cosmos sulphureus* extract inhibits the growth of *Cyperus rotundus*. Hossen et al. (2020) found that the aqueous extract of *Wedelia chinensis* significantly inhibited the germination and seedling growth of lettuce (*Lactuca sativa*), alfalfa (*Medicago sativa*), cress (*Lepidium sativum*), rapeseed (*Brassica napus*), Italian ryegrass (*Lolium multiflorum*), barnyard grass, foxtail fescue (*Vulpia myuros*) and timothy (*Phleum pratense*). Additionally, a more recent study by Bashar et al. (2023) indicated that *Parthenium hysterophorus* has significant allelopathic impacts on the germination and growth of V*igna subterranea*, *Raphanus sativus*, *Cucurbita maxima*, *Cucumis sativus*, *Solanum lycopersicum*, *Capsicum frutescens*, *Zea mays*, *Abelmoschus esculentus*, *Daucus carota*, *Digitaria sanguinalis*, *and Eleusine indica*. Furthermore, Maksimović et al. (2023) highlighted the allelopathic potential of *Ambrosia artemisiifolia*, showing that its extracts can significantly inhibit the growth of barley (*Hordeum vulgare*) and white clover (*Trifolium repens*). These findings underscore the potential of Asteraceae family plants as sources of natural herbicides, providing sustainable alternatives to synthetic chemicals.

Given the growing concerns over environmental sustainability and the adverse effects of synthetic herbicides, there is an urgent need to explore alternative weed management strategies. The Asteraceae family, known for its diverse allelopathic properties, offers promising potential as a source of natural herbicides. Despite this potential, there is a significant gap in the current literature regarding the phytotoxic effects of methanolic extracts from various Asteraceae species and their practical applications in weed control, especially for *Echinochloa crus‐galli* and *Echinochloa colona*. This study specifically addresses this gap by evaluating the phytotoxic potential of methanolic extracts from six Asteraceae species: *Wedelia chinensis*, *Helianthus annuus*, *Cosmos bipinnatus*, *Tagetes erecta*, *Tithonia diversifolia*, and *Zinnia elegans* on the two Echinochloa species. Using high‐performance liquid chromatography (HPLC), the active compounds in the methanolic extract of *C. bipinnatus* were identified. The findings from this research are expected to significantly advance the development of natural herbicides, contributing to more sustainable weed management practices.

## 2 MATERIALS AND METHODS

### 2.1 Plant materials

*Wedelia chinensis*, *Helianthus annuus*, *Cosmos bipinnatus*, *Tagetes erecta*, *Tithonia diversifolia*, *and Zinnia elegans* were collected from Vinh Long Province, Vietnam, at different growth stages. *E. crus‐galli* and *E. colona* were collected from experimental rice fields at the Cuu Long Delta Rice Research Institute, Can Tho, Vietnam.

### 2.2 Extraction of allelopathic methanolic extracts

All hypocotyls, leaves, and roots of the six Asteraceae species were cleaned thoroughly. Then, 40 g of mixed plant parts were prepared separately for Experiment 2 and at different growth stages for Experiment 3. The plant parts were cut into small pieces and immersed in 400 mL of 60% MeOH in a triangular flask. This mixture was soaked for 48 h.

After soaking, the extract was filtered using filter paper, and the first extract was stored in a refrigerator at 5–8°C. The remaining plant material was then subjected to a second extraction using 200 mL of 100% MeOH and soaked for another 48 h. Both extracts were combined and the MeOH solvent was evaporated using a rotary vacuum evaporator (Yamato Neocool Circulator CF302L, Yamato Rotary Evaporator RE301, Yamato Water Bath BM510, Yamato. T. Suzuki, Japan), resulting in 80 mL of a water‐based extract containing allelochemicals. The pH of the final extract was adjusted to 7.0 using a phosphate buffer.

### 2.3 Evaluation of inhibitory ability

The extract was placed in Petri dishes lined with filter paper, with varying concentrations (0.03, 0.1, 0.3, and 1.0 g fresh plant/mL). An additional concentration of 0.5 g fresh plant/mL was used for experiments involving different plant parts and growth periods of the most allelopathic Asteraceae species. The extract was then dried completely in a fume hood to remove any residual MeOH. The dried extracts on the filter papers were moistened with 1.0 mL of Tween 20 solution (0.05%) and 10 newly sprouted seeds of *E. crus‐galli* or *E. colona* were placed on the filter paper, covered by plastic and aluminum foils right after that, and incubated in darkness at 25°C. For the control treatment, seeds were sown on a blank filter paper with 1.0 mL of Tween 20 solution only (Thi et al., 2014). After 48 h, the hypocotyl and root lengths of *E. crus‐galli* and *E. colona* were measured. The plant growth‐suppressing efficiency was calculated using Abbott's formula (1925).

### 2.4 Determination of allelochemicals in Asteraceae plant part at 60 days old

High‐pressure liquid chromatography (HPLC) was utilized to analyze Asteraceae tissue extracts at 60 days old using a Shimadzu LC‐2030C HPLC system with a −10A VP module and CLASS‐VP software (Shimadzu Co., Ltd). The chromatographic separation was performed using a VertiSep™ GES C18 HPLC column (250 × 4.6 mm, 5.0 μm). The mobile phase consisted of methanolic (A) and water with 0.1% formic acid (B), using a gradient elution program. The analysis was conducted at a flow rate of 0.8 mL/min with UV detection from 200 to 400 nm. The characteristic absorbance peaks of target allelochemicals, including cinnamic acid, caffeic acid, coumaric acid, ferulic acid, 2,4‐dimethoxybenzoic acid, and salicylic acid, were identified and quantified.

### 2.5 Statistical analysis

The data were converted into % inhibition efficiency determined by the formula:

Inhibition efficiency R = (L1 – L2)/L1 × 100.

In which: R: inhibition efficiency (%); L1: average length of roots or shoots of control plants; L2: average length of roots or shoots of treated plants.

SPSS software (version 20.0) was used to analyze the data.

## 3 RESULTS

### 3.1 Inhibition capacity of methanolic extracts from six species of the Asteraceae family on the growth of *Echinochloa crus‐galli* and *Echinochloa colona*

**FIGURE 1:**
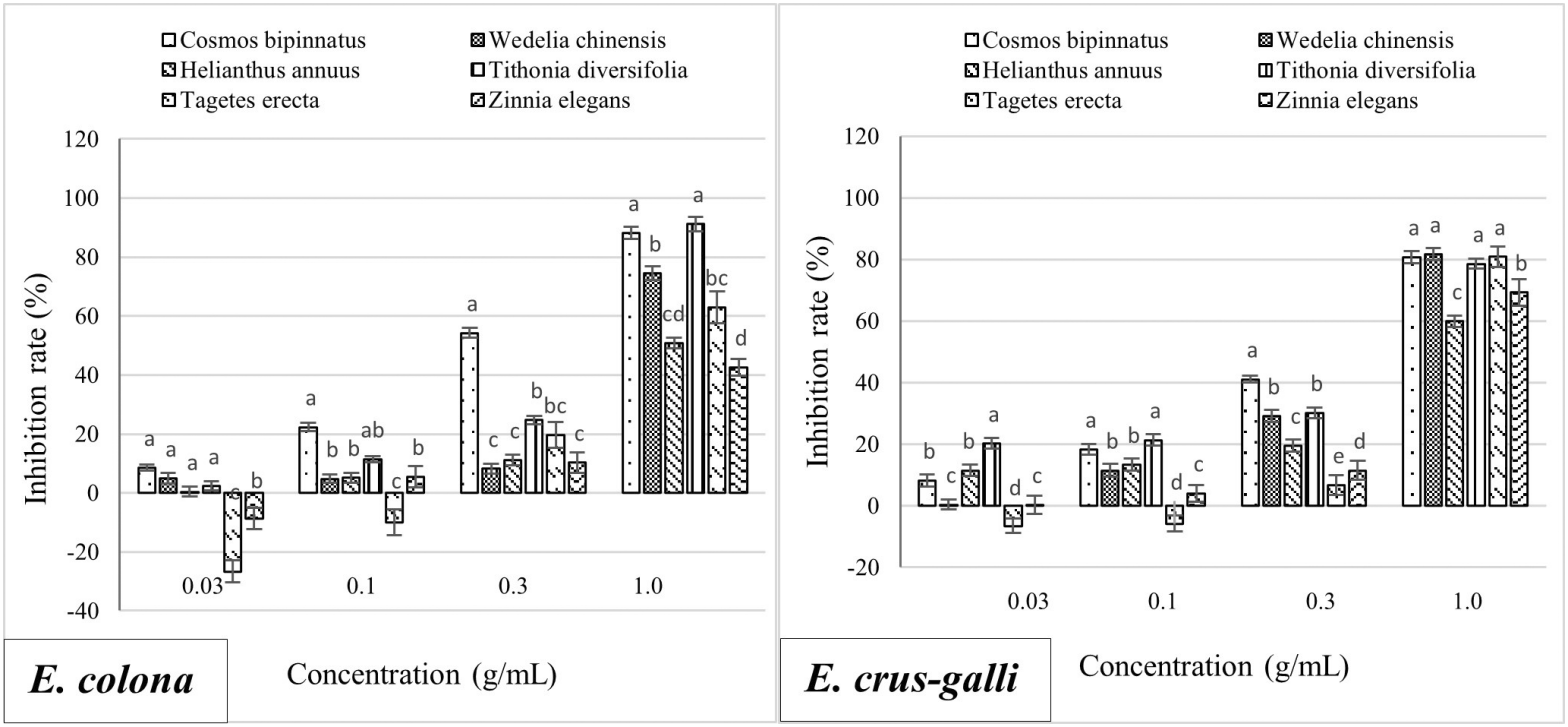
Inhibitory effect of extracts of six Asteraceae species on the hypocotyl length of *E. colona* (left) and *E. crus‐galli* (right). The concentrations of the tested samples corresponded to the extracts that were obtained from 0.03, 0.1, 0.3, and 1.0 g freshweight Asteraceae plants (combine roots, shoots, and leaves). The means ± standard error (SE) from four replications, with 10 tested plants for each replication, is shown. The vertical bar indicates the percentage of the SE in comparison with the control. The means followed by the same letter are not significantly different, using Duncan's multiple‐range test at *p* ≤ 0.1.

The hypocotyls of *E. crus‐galli* and *E. colona* were less affected by methanolic extracts from the six Asteraceae species at low concentrations (0.03 and 0.1 g/mL). Particularly, the methanolic extract of *T. erecta* stimulated the hypocotyl growth of *E. crus‐galli* and *E. colona* by 6.55% and 26.69% at 0.03 g/mL, respectively. At higher concentrations of 0.3 and 1.0 g/mL, the methanolic extract of *C. bipinnatus* showed the highest inhibition efficiency of 41.06% and 80.73% for *E. crus‐galli* and 54.23% and 88.15% for *E. colona*, respectively, which was significantly different from that of *W. chinensis*, *H. annuus*, *C. bipinnatus*, *T. erecta*, and *Z. elegans*. Additionally, the methanolic extract of *T. diversifolia* at a concentration of 1.0 g/mL inhibited the radicle growth of *E. colona* by 91.7%, which was not significantly different from that of *C. bipinnatus* (88.15%) (Figure 1).

**FIGURE 2:**
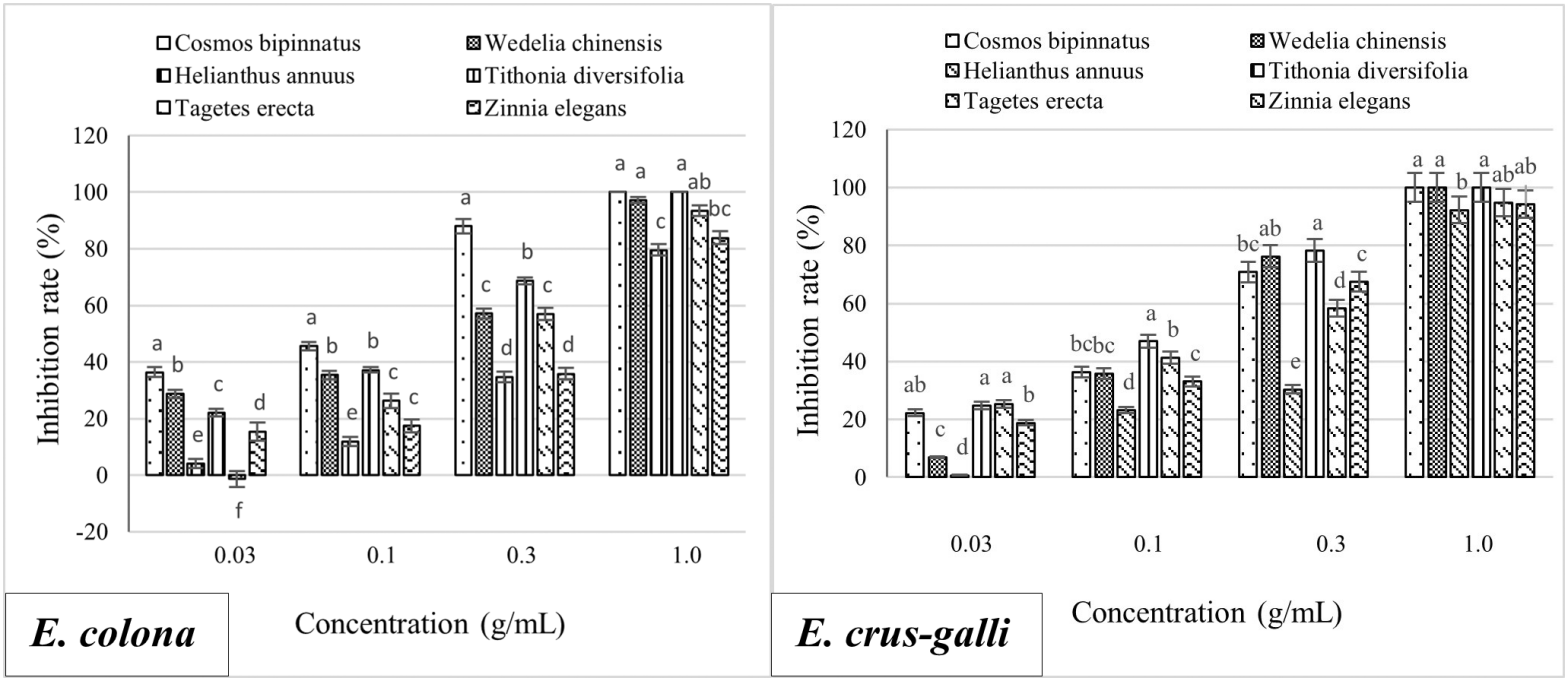
Inhibitory effect of extracts of six Asteraceae species on the radicle length of *E. colona* (left) and *E. crus‐galli* (right). The concentrations of the tested samples corresponded to the extracts that were obtained from 0.03, 0.1, 0.3, and 1.0 g freshweight Asteraceae plants (combine roots, shoots, and leaves). The means ± standard error (SE) from 4 replications, with 10 tested plants for each replication, are shown. The vertical bar indicates the percentage of the SE in comparison with the control. The means followed by the same letter are not significantly different, using Duncan's multiple‐range test at *p* ≤ 0.1.

**FIGURE 3:**
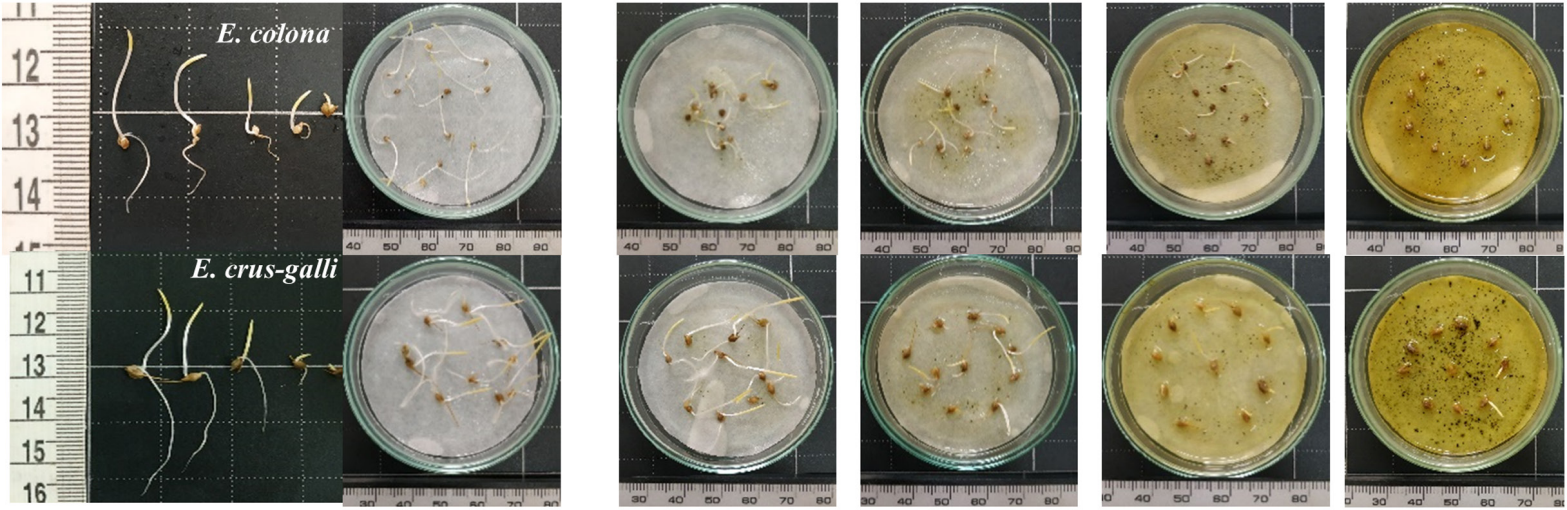
*E. colona* (top) and *E. crus‐galli* (bottom) are inhibited by the methanolic extract from *Cosmos bipinatus*, from left to right, the concentration gradually increases to 0, 0.03, 0.1, 0.3, and 1.0 g/mL.

The radicles of *E. crus‐galli* and *E. colona* were more sensitive to the methanolic extracts from the Asteraceae species than the hypocotyls. The low concentration (0.03 g/mL) methanolic extract of *C. bipinnatus* showed the highest inhibition efficiency of 20.37% and 36.35% on the radicle length of *E. crus‐galli* and *E. colona*, respectively, which was significantly different from that of other five Asteraceae species. At a concentration of 0.3 g/mL, the inhibitory efficiency on the radicle length of *E. crus‐galli* and *E. colona* was more evident, with the methanolic extract of *T. diversifolia* showing the highest inhibition efficiency of 78.17% for *E. crus‐galli* and that of *C. bipinnatus* showing the highest inhibition efficiency of 87.93% for *E. colona*, which were significantly different. At the highest concentration of 1.0 g/mL, the inhibitory efficiency of Asteraceae extracts on the radicle length of *E. crus‐galli* and *E. colona* was very high, and the methanolic extracts of *T. diversifolia* and *C. bipinnatus* inhibited the radicle growth of *E colona* almost 100%, significantly different from those of other species (Figure 2). The inhibitory efficiency of the Asteraceae methanolic extracts was proportional to the treatment concentration (Figure 3).

### 3.2 Inhibition ability of methanolic extract from different parts of *Cosmos bipinatus* and *Tithonia diversifolia* on the growth of *Echinochloa colona* and *Echinochloa crus‐galli*

**FIGURE 4:**
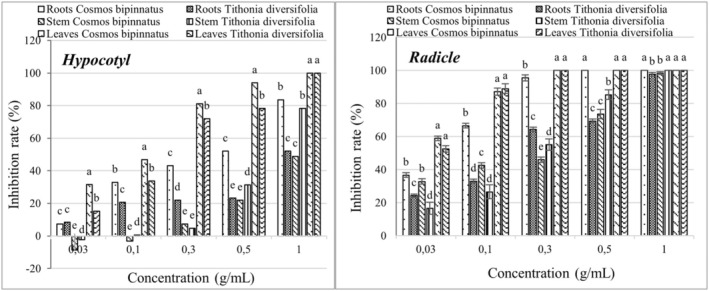
Inhibition effect of methanolic extract from different plant parts of *C. bipinatus* and *T. diversifolia* on the length of hypocotyl (left) and radicle (right) of *E. crus‐galli*. The concentrations of the tested samples corresponded to the extracts that were obtained from 0.03, 0.1, 0.3, 0.5, and 1.0 g freshweight plant part (roots, stems, and leaves separately). The means ± standard error (SE) from four replications, with 10 tested plants for each replication, is shown. The vertical bar indicates the percentage of the SE, in comparison with the control. The means followed by the same letter are not significantly different, using Duncan's multiple‐range test at *p* ≤ 0.1.

**FIGURE 5:**
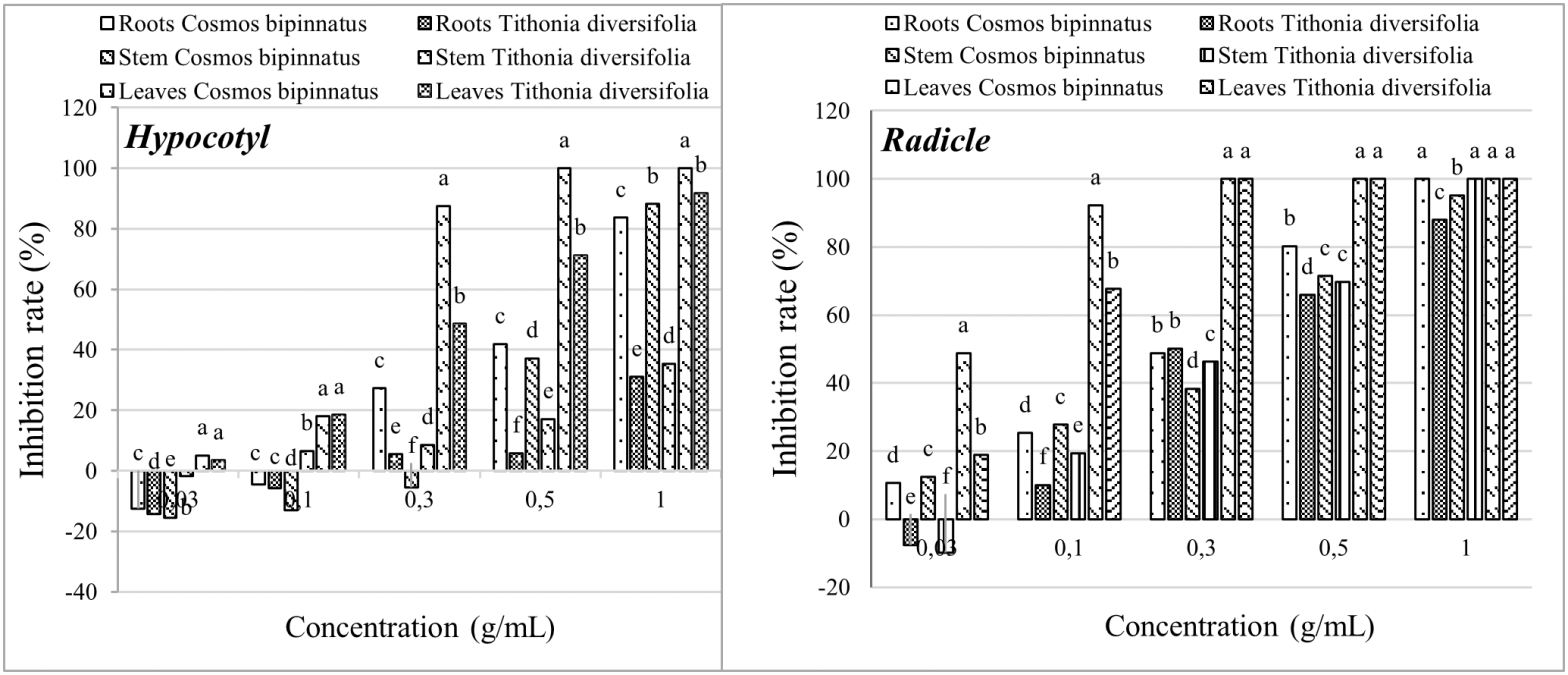
The inhibitory effect of the methanolic extract from different plant parts of *C. bipinatus* and *T. diversifolia* on the length of hypocotyl (left) and radicle (right) of *E. colona*. The concentrations of the tested samples corresponded to the extracts that were obtained from 0.03, 0.1, 0.3, 0.5, and 1.0 g freshweight plant parts (roots, stems, and leaves separately). The means ± standard error (SE) from four replications, with 10 tested plants for each replication, are shown. The vertical bar indicates the percentage of the SE, in comparison with the control. The means followed by the same letter are not significantly different, using Duncan's multiple‐range test at *p* ≤ 0.1.

**FIGURE 6:**
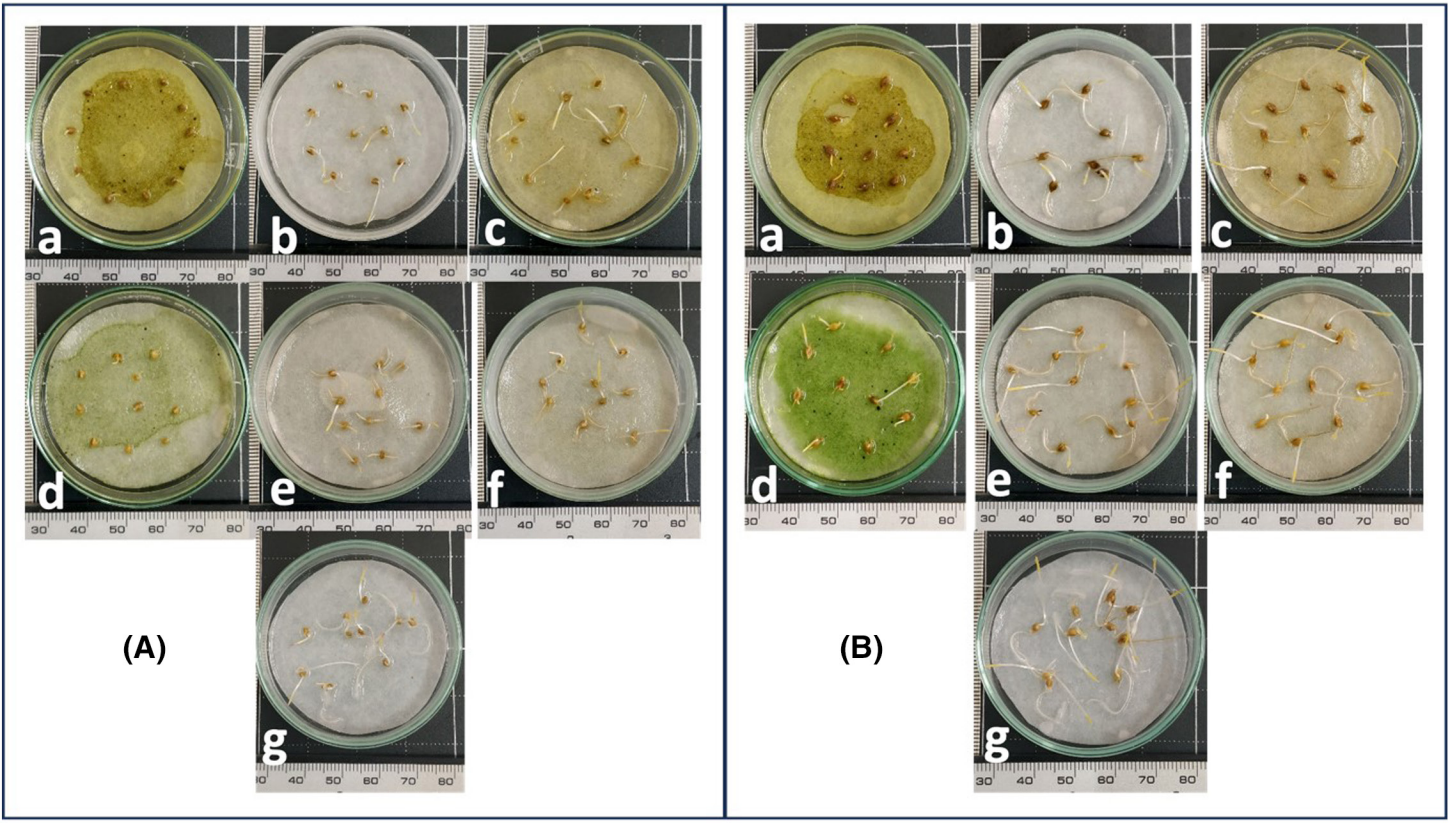
*E. crus‐galli* (A) and *E. colona* (B) are inhibited by methanolic extract from *C. bipinatus* and *T. diversifolia* leaves, stems, and roots at concentrations of 0.3 g/mL after incubating 48 hours in the dark. (a and d: Leaf of *C. bipinatus and T. diversifolia*; b and e: Root of *C. bipinatus* and *T. diversifolia*; c and f: Stem of *C. bipinatus* and *T. diversifolia*; *g*: No extract).

Based on the results of efficiency inhibition of six Asteraceae extracts on the hypocotyl and radicle of *E. crus‐galli* and *E. colona*, *C. bipinatus* and *T. diversifolia* were selected for further study to identify which plant part (stem/root/leaf) poses the highest weed growth inhibitory activity. In particular, the methanolic extract from the leaves of *C. bipinatus* showed the highest inhibition efficiency on the hypocotyl length of *E. crus‐galli*, respectively, to 31.49, 46.82, 71.89, 93.94, and 100% at concentrations of 0.03, 0.1, 0.3, 0.5, and 1.0 g/mL, which was significantly different from the extract from other parts of *C. bipinatus* and *T. diversifolia* (Figures 4 and 6). The radicles of *E. crus‐galli* were more sensitive to the methanolic extracts from the leaves of *C. bipinatus* and *T. diversifolia* at a concentration of 0.03 g/mL, achieving inhibitions of 58.91 and 52.49%, respectively, which were significantly different from the other treatments. The highest inhibition efficiency (100%) was achieved at a concentration of 0.3 g/mL for both plant extracts. Similar to *E. crus‐galli*, *E. colona* was also significantly inhibited by the methanolic extracts from the leaves of *C. bipinatus* and *T. diversifolia*. In this experiment, the leaf extract of *C. bipinatus* showed the highest inhibition efficiency on both the hypocotyl and radicle of *T. diversifolia*, which was significantly different from the other treatments (Figures 5 and 6). At a concentration of 0.3 g/mL, the leaf extract of *C. bipinatus* completely inhibited the radicle of *E. crus‐galli*.

The radicles of both *E. crus‐galli* and *E. colona* were significantly inhibited when exposed to methanolic extracts from the *C. bipinatus* and *T. diversifolia* materials at a concentration of 0.03 g/mL (58.91% and 48.78%, respectively), and the inhibition rate was proportional to the concentration of the treatment (Figure 6). The inhibition efficiency of the methanolic extracts from the stems and roots of *C. bipinatus* and *T. diversifolia* was lower than that of the leaf extracts. Among them, the stem extracts showed the lowest inhibition efficiency.

### 3.3 Inhibition ability of methanolic extract from the leaf of *Cosmos bipinatus* at different leaf ages on *Echinochloa colona* and *Echinochloa crus‐galli*

**FIGURE 7:**
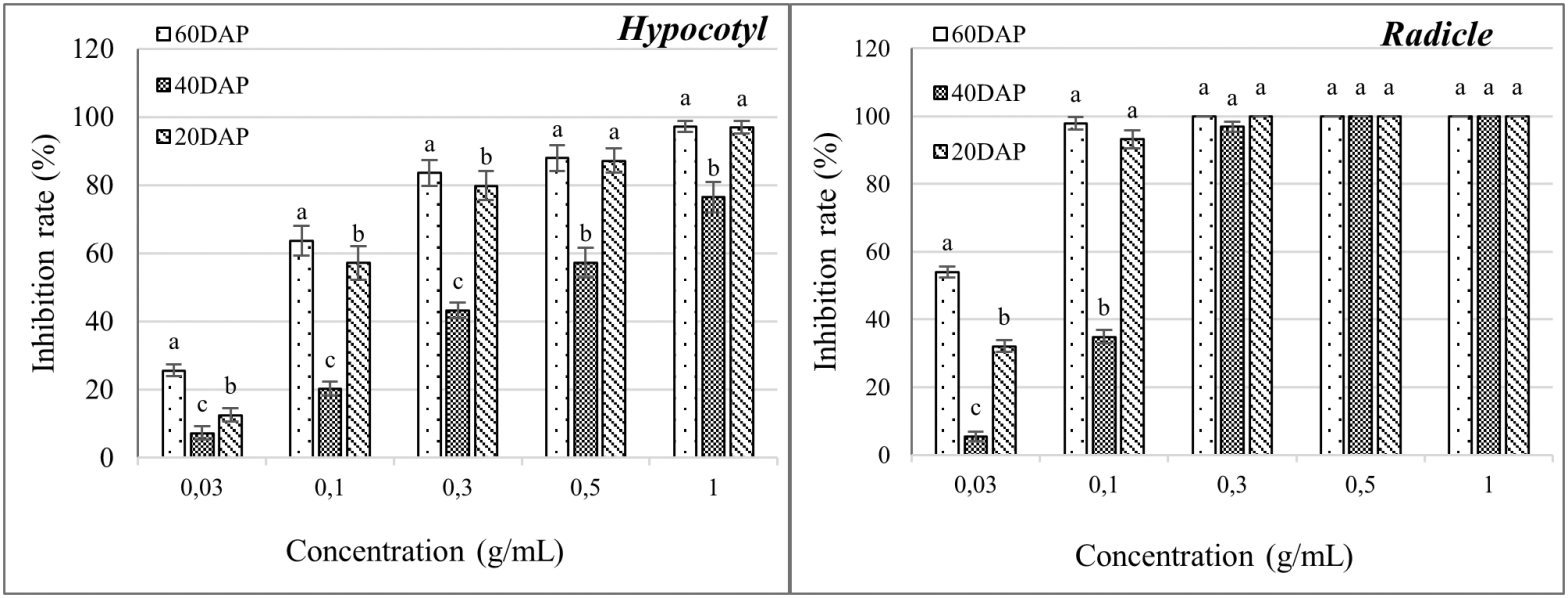
The inhibitory effect of the methanolic leaf extract of *C. bipinatus* at 20, 40, and 60 days after planting (DAP) on the length of hypocotyl (left) and radicle (right) of *E. colona*. The concentrations of the tested samples corresponded to the extracts that were obtained from 0.03, 0.1, 0.3, 0.5, and 1.0 g freshweight plant parts (roots, stems, and leaves separately). The means ± standard error (SE) from four replications, with 10 tested plants for each replication, are shown. The vertical bar indicates the percentage of the SE in comparison with the control.

**FIGURE 8:**
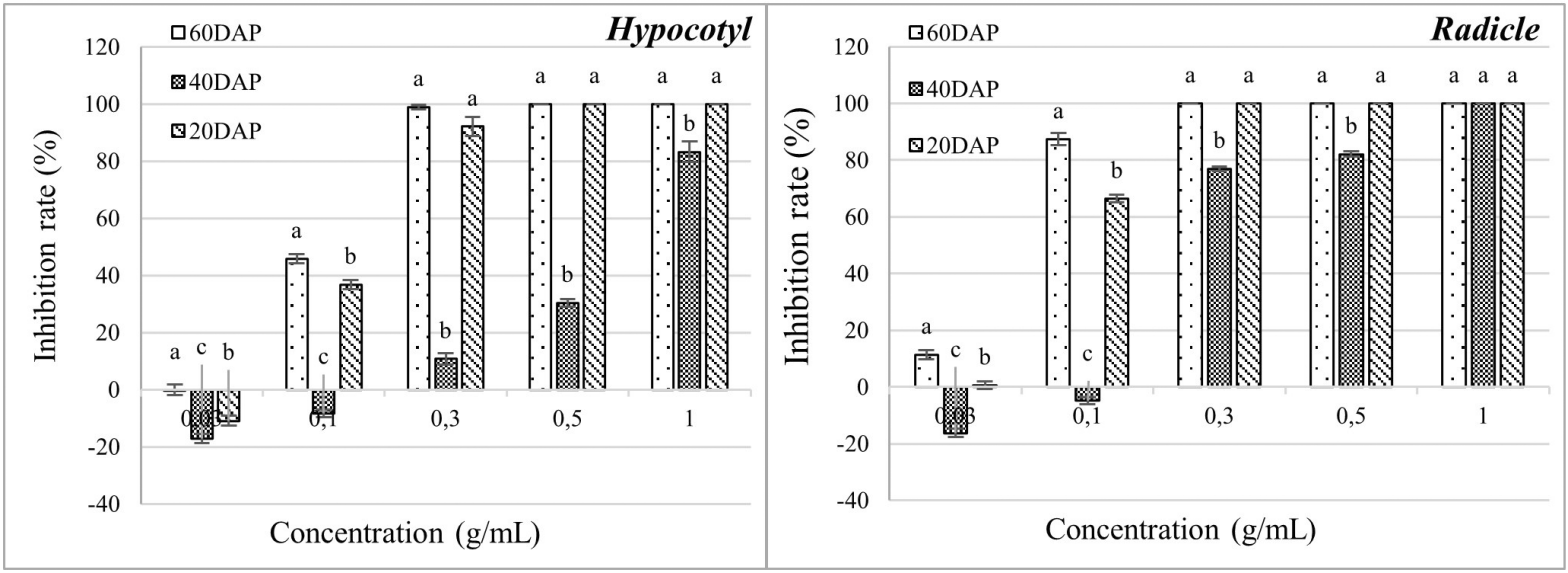
The inhibitory effect of the methanolic leaf extract of *C. bipinatus* at 20, 40, and 60 days after planting (DAP) on the length of hypocotyl (left) and radicle (right) of *E. crus‐galli*. The concentrations of the tested samples corresponded to the extracts that were obtained from 0.03, 0.1, 0.3, 0.5, and 1.0 g freshweight plant parts (roots, stems, and leaves separately). The means ± standard error (SE) from four replications, with 10 tested plants for each replication, are shown. The vertical bar indicates the percentage of the SE, in comparison with the control.

**FIGURE 9:**
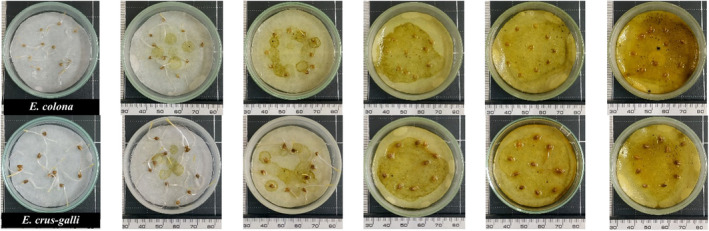
*E. colona* (above) and *E. crus‐galli* (below) inhibited by the methanolic 60‐day‐old *C. bipinatus* leaf extract at the concentrations of 0.0, 0.03, 0.1, 0.3, and 1.0 g/mL from left to right.

The leaf of *C. bipinatus* showed the highest inhibitory activity on *E. crus‐galli* and *E. colona* among all plant parts of *C. bipinatus* and *T. diversifolia*. Therefore, it was researched further to find out which growth stage of *C. bipinatus* is the most responsible for the inhibitory activity. Similar to previous bioassay experiments, the radicles of *E. crus‐galli* and *E. colona* were more susceptible to inhibition by the extracts of *C. bipinatus* than the hypocotyls. The methanolic extract from 60‐day‐old *C. bipinatus* leaf was the most effective in inhibiting the growth of both radicles and hypocotyls of *E. crus‐galli* and *E. colona* (Figures 7, 8, 9), while the 40‐day‐old leaf showed lower inhibitory activity than the 60‐day‐old and 20‐day‐old leaves. The trend of inhibitory activity of the methanolic *C. bipinatus* leaf extract on both radicles and hypocotyls of *E. crus‐galli* and *E. colona* was 60‐ > 20‐ > 40‐day‐old, with significant differences.

The methanolic leaf extract of *C. bipinatus* at 60‐day‐old was most effectively inhibited the growth and development of *Echinochloa* sp. compared to other extracts. At a concentration of 0.3 g/mL, it completely inhibits the radicles of the weed species. The 60‐day‐old *C. bipinatus* leaf extract shows inhibitory efficacy on *E. colona* at the lowest concentration (0.03 g/mL) at 54.06% and almost complete inhibition (97.85%) at a concentration of 0.1 g/mL. For *E. crus‐galli*, it inhibits the radicles by 87.32% at a concentration of 0.1 g/mL (Figures 7, 8, 9).

### 3.4 Allelochemical composition in 60‐day‐old *Cosmos bipinatus* leaf extract

Based on the experimental results of the inhibitory effects of the methanolic 60‐day‐old *C. bipinatus* leaf extract on hypocotyl and radicle growth of *Echinochloa* sp., we selected 60‐day‐old *C. bipinatus* leaf extract to determine the allelochemicals using HPLC.

**FIGURE 10:**
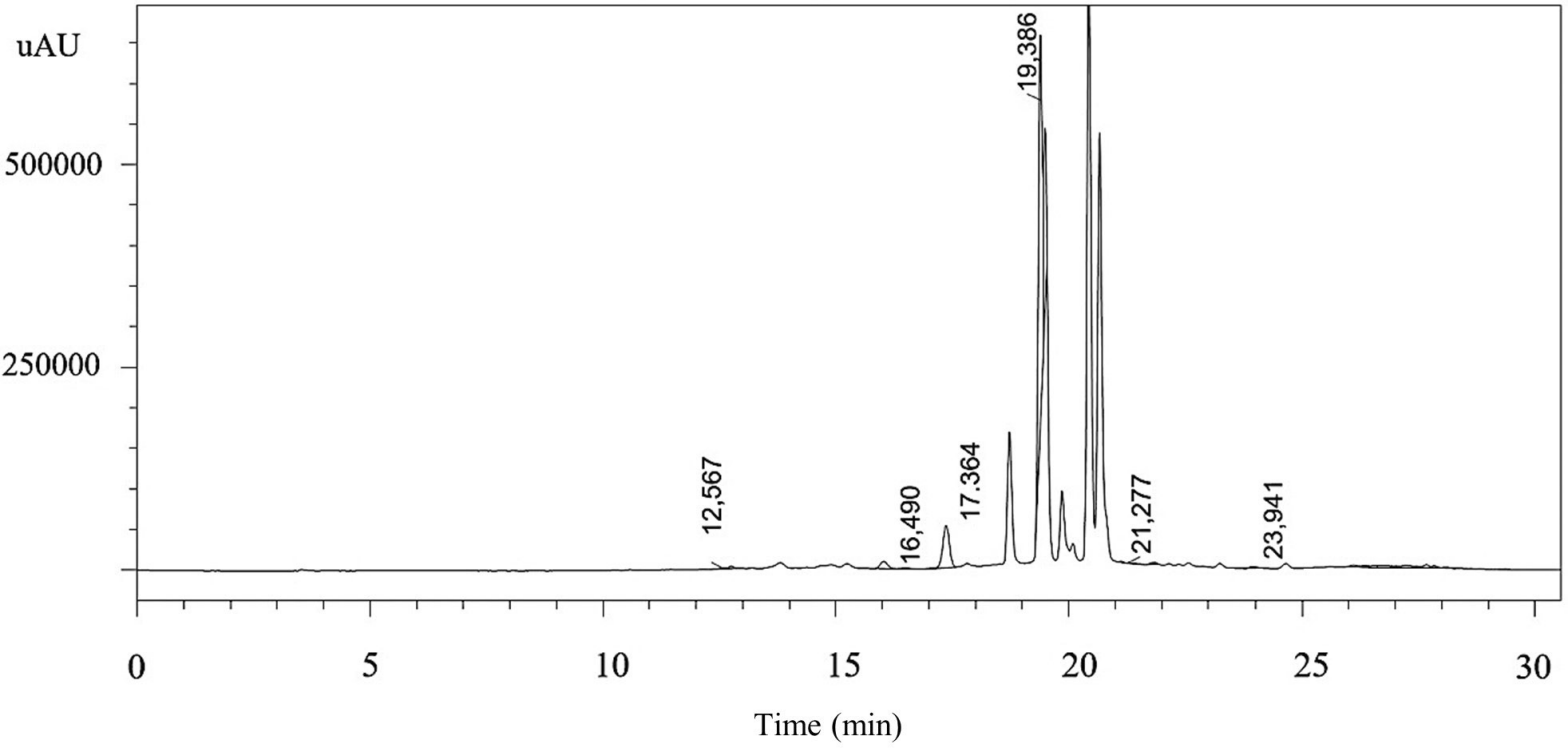
The HPLC peaks related to the identified allelochemicals: Caffeic acid (12.567 min); coumaric acid (16.490 min); ferulic acid (17.364 min); 2‐4 dimethohydroxy benzoic (19.386 min); salicylic acid (21.277 min); and cinnamic acid (23.941 min).

Phenolic and ester compounds are common secondary metabolites with weed‐suppressing activity. Through the experiment evaluating the inhibitory ability from the extract of *C. bipinatus* leaves collected at the age of 20, 40, and 60 days, 60‐day‐old *C. bipinatus* leaf extract was used to determine the composition of antagonists using HPLC high‐performance liquid chromatography. The results showed that a number of plant antagonistic compounds in 60‐day‐old *C. bipinatus* leaf belonging to phenolic and ester groups were found after analysis by HPLC at 12.567 (caffeic acid), 16.490 (coumaric acid), 17,364 (ferullic acid), 19,386 (2‐4 dimethohydroxy benzoic), 21,277 (salicylic acid), and 23,941 min (cinamic acid) (Figure 10; Table 1), corresponding to the contents (mg/L) listed in Table 1 such as: caffeic acid (0.054), coumaric acid (0.1), ferullic acid (8.005), 2‐4 dimethohydroxy benzoic (157.121), salicylic acid (0.276), and cinamic acid (0.292).

**TABLE 1:** The content of allelochemicals in 60‐day‐old *Cosmos bipinatus* leaf.

Allelochemicals	Retention time (RT)	Peak area	Peak height	Content in sample (mg/L)	Content in leaf (μg/g)
Cinnamic acid	23.941	37.175	2.820,00	0.292,00	584,00
Caffeic acid	12.567	5.475	599,00	0.054,00	108,00
Coumaric acid	16.490	10.629	942,00	0.100,00	200,00
Ferulic acid	17.364	510.813	51.912,00	8.005,00	16.010,00
2‐4 Dimethohydroxy benzoic	19.386	2.813.896	483.132,00	157.121,00	314.242,00
Salicylic acid	21.277	5.071	327,00	0.276,00	552,00

*Note*: The volume of 60‐day‐old Cosmos bipinatus leaf extract (V = 600 mL) corresponds to 100 g of fresh 60‐day‐old Cosmos bipinnatus leaves; 1 mL of extract contains 0.167 g of fresh 60‐day‐old Cosmos bipinatus leaves.

## 4 DISCUSSION

Allelopathy, a phenomenon where plants release bioactive compounds into their environment, plays a crucial role in plant–plant interactions and can influence the composition and structure of plant communities (Einhellig, 1995; Rice, 1984). Understanding the underlying mechanisms of allelopathy is imperative for harnessing the full potential of Asteraceae plants in weed management strategies. The research findings presented in this study unveil the remarkable allelopathic potential of six Asteraceae plant species, including *W. chinensis*, *H. annuus*, *C. bipinnatus*, *T. erecta*, *T. diversifolia*, and *Z. elegans*, as effective inhibitors of weed growth weeds and can be used as raw materials to produce bioherbicides. These results are consistent with previous studies suggesting the presence of allelopathic compounds in Asteraceae plants, which significantly contribute to their weed‐suppressing properties (Araújo et al., 2021; Hossen et al., 2020; Laosinwattana et al., 2018; Respatie et al., 2019). The *T. diversifolia* and *W. chinensis* pose allelopathic potential in this study, which has been demonstrated by Nie et al. (2004), Oyerinde et al. (2009), Rensen et al. (1994), Tongma et al. (1998) and Hossen et al. (2020). Although *H. annuus* has been shown to have allelopathic potential (Abeysekera & Robles, 1993; Anjum & Bajwa, 2010; Bogatek et al., 2006; Cholid, 2004), its allelopathic potential in this study is lower compared to other five Asteraceae species.

The methanolic extract of *C. bipinnatus* leaves contains plant allelopathic substances that can greatly inhibit the hypocotyl and radicle growth of two types of weeds, *E. colona and E. crus‐galli*. Studies by Céspedes et al. (2006), Mata et al. (2002) and Respatie et al. (2019) have supported our research findings on the greatest allelopathic potential of *C. bipinnatus* on some tested plant species. The radicles of *E. crus‐galli* and *E. colona* exhibited greater sensitivity to the methanolic extracts from *C. bipinnatus* compared to the hypocotyls. Wu et al. (2009) also concluded that the leaf extract of *C. bipinatus* had higher efficiency inhibition than the root extract. Moreover, other studies have also shown that leaf is the plant part with higher allelopathic efficiency than root (Asgharipour & Armin, 2010; Zhang et al., 2010).

The extract from *C. bipinnatus* 60‐day‐old leaves showed higher inhibition compared to extracts from 40‐day‐old and 20‐day‐old leaves. The identified compounds in the extract from *C. bipinnatus* 60‐day‐old leaves include caffeic acid, coumaric acid, ferulic acid, 2‐4 dimethohydroxy benzoic, salicylic acid, and cinnamic acid. Therefore, the leaves of *C. bipinnatus* at this stage are a potential source of plant‐based herbicides to control weeds in the rice fields, particularly *E. colona* and *E. crus‐galli*. The inhibitory activity of methanolic *C. bipinatus* leaf extract on both radicles and hypocotyls of *E. crus‐galli* and *E. colona* showed a distinct trend, with the most potent effects observed at 60 days, followed by 20 and 40 days, demonstrating significant differences. This aligns with findings by Abdel‐Farid et al. (2007), who reported higher flavonoid allelopathic compound concentrations in 4‐week‐old cabbage plants compared to 6‐week‐old plants, and with Mediani et al. (2012), who noted peak phenolic content in *C. bipinatus* at 8 weeks of age. These studies indicate that *C. bipinnatus* has a high potential as a plant allelopathic agent and can be used as a natural herbicide to control weeds.

One of the key aspects highlighted in this study is the importance of optimizing extraction methods to enhance the recovery of allelopathic compounds from Asteraceae plants. Different extraction techniques, such as solvent extraction, steam distillation, or supercritical fluid extraction, can be explored to ensure the maximum yield of bioactive compounds (Sharma et al., 2019). By optimizing extraction protocols, allelochemicals can be effectively isolated and characterized, facilitating further investigations into their mechanisms of action and potential applications in weed control. Furthermore, identifying the optimal concentration and dosage of allelochemicals derived from Asteraceae plants is essential for developing effective weed management strategies while minimizing adverse effects on non‐target organisms (Li et al., 2019; Scavo & Mauromicale, 2020). Dosage optimization studies enable researchers to determine the concentration thresholds required for weed suppression while ensuring the safety of surrounding crops and ecosystems. Additionally, understanding the interactions between allelochemicals and target weed species can provide valuable insights into the development of selective and eco‐friendly herbicides (Wu et al., 2024).

The methanolic extract of *C. bipinnatus* leaves emerged as a potent inhibitor of hypocotyl and radicle growth in both *E. colona* and *E. crus‐galli*, underscoring its potential as a natural herbicide. This finding is consistent with previous studies that have highlighted the allelopathic properties of Asteraceace species extracts (La Iacona et al., 2024; Lopes et al., 2022). Chemical analysis of the extract revealed the presence of several allelopathic compounds, including caffeic acid, coumaric acid, ferulic acid, 2‐4 dimethohydroxy benzoic, salicylic acid, and cinnamic acid. These compounds are known to interfere with various physiological and biochemical processes in weed species, ultimately leading to growth inhibition (Nkomo et al., 2019; Thi et al., 2022).

Evaluating the allelopathic potential and mechanisms of identified compounds as plant growth suppression inhibitors provides valuable insights into their mode of action and efficacy in weed management. Allelopathic compounds interfere with various physiological and biochemical processes in target weed species, ultimately leading to growth inhibition and reduced competitive ability (Khanh et al., 2005). Caffeic acid, coumaric acid, ferulic acid, 2‐4 dimethohydroxy benzoic acid, salicylic acid, and cinnamic acid are among the allelopathic compounds identified in the methanolic extract of *C. bipinnatus* leaves. These compounds have been extensively studied for their allelopathic properties and mechanisms of action. For example, caffeic acid has been shown to inhibit seed germination and root growth by disrupting cell division and inducing oxidative stress in target weed species (Chen et al., 2022; Pan et al., 2023). According to Batish et al. (2008), caffeic acid interfered with the rooting ability of mung bean hypocotyl cuttings by changing the activities of peroxidase (POD), polyphenol oxidase (PPO) and total endogenous phenol (TP) content, plays a key role in the rooting process. Similarly, coumaric acid interferes with root elongation and cell division processes, leading to growth inhibition in weeds (Chon et al., 2002), it has also been shown to have a serious effect on soybean root growth (Einhellig & Eckrich, 1984). Caffeic acid, coumaric acid, ferulic acid, and cinnamic acid at concentrations ranging from 10 to 30 μmol/L inhibit the growth of soybean (*Glycine max*) (Patterson, 1981). Ferulic acid has been reported to inhibit photosynthesis and disrupt cellular metabolism in weed species, resulting in reduced growth and biomass accumulation (Farooq et al., 2009). Furthermore, salicylic acid and p‐coumaric acid at a concentration of 10 mM significantly inhibited the growth of plants such as wavy‐hair grass (*Deschampsia flexuosa*), figwort (*Scrophularia nodosa*), and woodland ragwort (*Senecio sylvaticus*), and fireweed (*Chamaenerion angustifolium*) (Nkomo et al., 2019). Salicylic acid acts as a signaling molecule that regulates plant defense responses and inhibits weed growth by inducing systemic acquired resistance and oxidative stress in target plants (Pokotylo et al., 2022; Thi et al., 2022). Cinnamic acid exhibits phytotoxic effects by disrupting cell membrane integrity and inhibiting enzyme activities essential for plant growth and development (Singh & Amist, 2018). At a concentration of 2.5 mM, 2,4‐dimethoxybenzoic acid inhibited the growth of shoots and roots of *E*. *colona* at 68.1% and 100%, respectively (Thi et al., 2022). The composition of compounds identified in the 60‐day‐old *C. bipinatus* leaves provides a clear explanation for the observed effects of the methanolic extract from these leaves on the growth and development of *E. crus‐galli* and *E. colona* at different concentrations. These allelopathic compounds target multiple biochemical pathways and physiological processes in weed species, making them effective inhibitors of weed growth and proliferation.

Allelopathy and allelochemicals hold significant promise for practical applications in crop production, particularly in the development of bioherbicides—natural products used for weed control. Unlike traditional herbicides, which are often synthetic chemicals, bioherbicides derive from natural sources and include phytotoxic plant‐based secondary metabolites (Cordeau et al., 2016). This reality underscores the growing interest in leveraging allelopathic interactions to enhance sustainable agricultural practices. However, despite the potential of allelopathy, most current bioherbicides in the market are based on fungal or bacterial microorganisms, with only a few incorporating natural plant extracts. For example, a product containing the active ingredient pelargonic acid, naturally found in various vegetables and fruits, partially controls broadleaf and grass weeds (Ciriminna et al., 2019). However, pelargonic acid itself is not classified as an allelochemical. In fact, a notable bioherbicide based on allelochemicals is “sorgaab,” which is a water extract of mature sorghum plants. This extract effectively inhibits weed growth in wheat fields (Cheema et al., 2008; Cheema & Khaliq, 2000; Głąb et al., 2017). Additionally, a patent registered in South Korea in 2005 for rice momilactones A and B highlights another promising avenue (Patent: KR20060083774A). These compounds have been commercially developed and demonstrated for the potential of allelochemicals in practical weed management (Zhao et al., 2018). Therefore, focusing on plant extract with growth‐suppressive characteristics with the aim to identify compounds with structural similarities that enhance their efficacy. Concurrently, exploring plant extracts with inherent allelochemical properties remains a critical strategy in developing effective bioherbicides for modern agriculture.

In conclusion, the allelopathic potential of the extracts from six Asteraceae plant species, including *W. chinensis*, *H. annuus*, *C. bipinnatus*, *T. erecta*, *T. diversifolia*, and *Z. elegans*, as effective inhibitors of weed growth, further emphasizes the importance of exploring natural solutions for weed control. The identification and characterization of allelopathic compounds from *C. bipinnatus* provide valuable insights into their potential as natural herbicides for weed management. Understanding the mechanisms of action of these compounds is essential for optimizing their utilization in sustainable weed control strategies. Further research is warranted to elucidate the specific biochemical pathways targeted by allelopathic compounds and their long‐term effects on weed populations and agroecosystems such as: conduct detailed studies to understand the biochemical pathways targeted by the allelopathic compounds identified in *c. bipinnatus* and other Asteraceae species, analyzing how these compounds interfere with weed growth processes; perform experiments to determine the optimal concentrations of allelopathic extracts for effective weed inhibition without harming crops, establishing a reliable application rate for implementing field trials to evaluate the practical effectiveness of these extracts in real agricultural settings, assessing their impact on weed suppression, crop yield, and soil health over multiple growing seasons; conduct assessments to ensure these natural herbicides do not negatively affect non‐target organisms, promoting ecological balance and sustainability; develop formulations and delivery systems to maximize the stability and efficacy of the allelopathic compounds, such as encapsulation techniques and slow‐release formulations; and work toward obtaining regulatory approval and focus on farmer education and adoption by demonstrating the benefits, safety, and ease of use of these new weed management strategies. Following this pathway ensures a systematic evaluation of the allelopathic potential of Asteraceae species for sustainable weed management, bridging the gap between laboratory research and practical application, and ultimately contributing to eco‐friendly farming practices.

## CONFLICT OF INTEREST STATEMENT

The authors declare no conflict of interest.

## Data Availability

The datasets generated and analyzed during the current study are available in the figshare repository: https://doi.org/10.6084/m9.figshare.26309764.v1.
